# Ethylene promotes anthocyanin synthesis in ‘Viviana’ lily via the LvMYB5-LvERF113-LvMYB1 module

**DOI:** 10.1093/hr/uhaf059

**Published:** 2025-02-25

**Authors:** Yibing Zhang, Yibo Sun, Weifeng Du, Shaokun Sun, Shimiao Zhang, Mengyao Nie, Yudong Liu, Muhammad Irfan, Li Zhang, Lijing Chen

**Affiliations:** Key Laboratory of Agriculture Biotechnology, Key Laboratory of Protected Horticulture (Ministry of Education), College of Biosciences and Biotechnology, Shenyang Agricultural University, No. 120 Dongling Road, Shenhe District, Shenyang, Liaoning 110161, China; Key Laboratory of Agriculture Biotechnology, Key Laboratory of Protected Horticulture (Ministry of Education), College of Biosciences and Biotechnology, Shenyang Agricultural University, No. 120 Dongling Road, Shenhe District, Shenyang, Liaoning 110161, China; Key Laboratory of Agriculture Biotechnology, Key Laboratory of Protected Horticulture (Ministry of Education), College of Biosciences and Biotechnology, Shenyang Agricultural University, No. 120 Dongling Road, Shenhe District, Shenyang, Liaoning 110161, China; Institute of Vegetable Research, Liaoning Academy of Agricultural Sciences, No. 84 Dongling Road, Shenhe District, Shenyang, Liaoning 110161, China; Key Laboratory of Agriculture Biotechnology, Key Laboratory of Protected Horticulture (Ministry of Education), College of Biosciences and Biotechnology, Shenyang Agricultural University, No. 120 Dongling Road, Shenhe District, Shenyang, Liaoning 110161, China; Key Laboratory of Agriculture Biotechnology, Key Laboratory of Protected Horticulture (Ministry of Education), College of Biosciences and Biotechnology, Shenyang Agricultural University, No. 120 Dongling Road, Shenhe District, Shenyang, Liaoning 110161, China; Key laboratory of Special Fruits and Vegetables Cultivation Physiology and Germplasm Resources Utilization Xinjiang of Production and Construction Crops, College of Agriculture, Shihezi University, No. 221, Beisi Road, Shihezi City, Xinjing 832003, China; Department of Biotechnology, University of Sargodha, Sargodha 40100, Pakistan; Key Laboratory of Agriculture Biotechnology, Key Laboratory of Protected Horticulture (Ministry of Education), College of Biosciences and Biotechnology, Shenyang Agricultural University, No. 120 Dongling Road, Shenhe District, Shenyang, Liaoning 110161, China; Key Laboratory of Agriculture Biotechnology, Key Laboratory of Protected Horticulture (Ministry of Education), College of Biosciences and Biotechnology, Shenyang Agricultural University, No. 120 Dongling Road, Shenhe District, Shenyang, Liaoning 110161, China

## Abstract

Ethylene (ET) influences the synthesis of anthocyanins, although its regulatory effects can differ significantly across various plant species. In apples (*Malus domestica*), ET promotes anthocyanin synthesis, whereas in *Arabidopsis thaliana*, it inhibits its accumulation. Our research showed that ethephon (Eth), an ET derivative, promotes anthocyanin synthesis in ‘Viviana’ lilies, which has great potential in the cut flower industry. The regulatory mechanism whereby ET influences anthocyanin synthesis in lilies remains unclear. In this study, we screened and characterized an ET-induced ET response factors (ERFs), LvERF113, with inhibitory function. Our analyses suggested that LvERF113 could inhibit the negative regulatory function of LvMYB1 at transcriptional and posttranslational levels, promoting anthocyanin synthesis in ‘Viviana’ lily tepals. In addition, LvERF113 is positively regulated by LvMYB5, forming the LvMYB5-LvERF113-LvMYB1 module controlling anthocyanin synthesis by ET in ‘Viviana’ lily. These findings offer new insights into the ET regulatory network of anthocyanin synthesis and provide a theoretical basis for the application of ET derivatives in the cut flower industry.

## Introduction

The modern flower industry is environmentally friendly, providing ecological, economic, and social benefits. It is important in both developed and underdeveloped countries [1]. The cultivation area and production of cut flowers, potted plants, and ornamental seedlings has increased over time, with cut flowers being one of the largest globally produced commercial items [2].

Flower color, a critical ornamental trait of cut flowers, directly determines their commercial value. Anthocyanins are a group of water-soluble flavonoid compounds present in plants and involved in various life processes, from the production of colorful spring flowers to autumn leaves; they are responsible for flower, fruit, and even leave colors from red to purple [3, 4]. Phenylalanine is a direct precursor, which undergoes sequential enzymatic reactions to generate anthocyanins and other flavonoids [5]. In this synthesis pathway, various structural and regulatory genes exert influence [6, 7]. Structural genes encode synthetic enzymes, directly modulating anthocyanin biosynthesis, including the early synthesis genes *chalcone synthase* (*CHS*), *chalcone isomerase* (*CHI*), *flavanone 3-hydroxylase* (*F3H*), and *flavonoid 3′-hydroxylase* (*F3′H*), and the late synthesis genes *dihydroflavonol-4-reductase* (*DFR*), *anthocyanin synthase* (*ANS*), and *flavonoid-3-O-glucosyltransferase* (*UFGT*) [8, 9]. MYB transcription factors (MYBs) are core regulatory elements affecting flower color [10, 11]. The MYBs involved in the regulation of anthocyanin synthesis are primarily R2R3-MYBs belonging to subgroup IV (SG4) and subgroup VI (SG6), which contain conserved DNA-binding domains in the R2 domain and bHLH-binding domains in R3 [12]. SG6 R2R3-MYBs contain a conserved activation structural domain and exert an activating function [13] while SG4 R2R3-MYBs contain the EAR motif (LxLxL or DLNxxP), tending to act as an indirect inhibitor in the anthocyanin synthesis network [14, 15] by competing for binding to bHLH proteins with the SG6 R2R3-MYBs in the MBW complex or directly inhibit structural gene expression by binding to their promoters [16–18].

As a critical phytohormone, ethylene (ET) is associated with various regulatory pathways, including plant growth and development [19, 20], secondary metabolite biosynthesis [21], and response to abiotic stress [22, 23]. Given their influence on fruit ripening and flower development, Eth and preservatives (ET inhibitors) are commonly employed in agricultural production strategies like fruit preservation during transportation and ripening for sale, flowering time control, and delayed flower senescence of cut flowers. While influencing fruit ripening and flowering time, ET impacts anthocyanin synthesis. Preservatives delay flower senescence while reducing the anthocyanin levels in rose petals [24]. Notably, in the anthocyanin synthesis pathway, ET has flexible regulatory features and may have opposite roles across plant tissues, or even in different parts of the same plant. For example, ET inhibits anthocyanin synthesis in seedlings and hypocotyls in *Arabidopsis* [25, 26], and ‘Hongzaosu’ pear peel [27, 28], but plays an activating role in apple and mango peel, and ‘Summer Blood Brine’ pear flesh [29–31].

In addition to directly modulating R2R3-MYBs, ET can regulate anthocyanin synthesis via ET response factor (ERFs). ERFs belong to the AP2/ERF family and contain the conserved AP2 domains, which can directly bind to promoter *cis*-elements such as GCC-box (GCCGCC) or DRE/CRT (CCGAC) [32]. By interacting with TPL proteins, some negative regulatory ERFs with EAR motifs inhibit downstream gene transcription [33]. Research has demonstrated that ERFs may regulate anthocyanin synthesis via three routes. (1) Directly regulate the expression of SG4 and SG6 R2R3-MYBs. PpERF105 directly activates the transcription of *PpMYB140* (SG4) [27], while PpERF9 directly inhibits the transcription of *PpMYB114* (SG6) to inhibit anthocyanin synthesis in pears [28]. (2) Interact with R2R3-MYBs to promote its binding ability to the structural gene promoters. MdERF38 physically interacts with MdMYB1 and facilitates MdMYB1 binding to the promoters of structural genes in apples [34]. (3) Directly bind to the structural gene promoters and induce their transcription. MdERF109 enhances apple anthocyanin content by directly regulating the expression of *MdCHS* and *MdUFGT* [35]. Although there are many studies on the function of ERFs, whether they can regulate anthocyanin synthesis in other ways remains to be further investigated.

Lily (*Lilium* spp.) is a perennial bulbous plant with graceful, upright, and elegant flowers. It is widely employed in cut flower arrangements and occupies a stable position in the flower market [36]. Early studies on the splatter-type spot formation of Asiatic hybrid lilies have shown that R2R3-MYB plays a crucial role in anthocyanin synthesis and accumulation in lily tepals [37, 38]. ‘Viviana’ lily belongs to the Oriental hybrid lily. In our previous research, two anthocyanin synthesis-related MYBs, LvMYB1 and LvMYB5, were cloned from ‘Viviana’ lily; they belong to the SG4 and SG6 subgroups of the R2R3-MYB subfamily and are involved in the regulation of anthocyanin synthesis in lily tepals as inhibitor and activator, respectively [39]. However, their regulatory and interaction relationships remain unclear. ET accelerates the flower maturation process of lilies, but its regulatory results in the flower coloration of Asiatic hybrid lilies and ‘Viviana’ lilies are different. ET inhibits anthocyanin synthesis in the tepals of Asiatic hybrid lilies [40, 41], while it exhibits the opposite effect in ‘Viviana’ lily tepals. The regulatory mechanism differences of ET on anthocyanin synthesis in the two lilies are still unclear.

In this study, we screened an ERF inhibitor, LvERF113, that mediates the ET-positive regulation of anthocyanin synthesis. We investigated the regulatory mechanism of LvERF113 and constructed a transcriptional cascade module, LvMYB5-LvERF113-LvMYB1. Our results reveal a regulatory pattern of ET-induced anthocyanin biosynthesis in ‘Viviana’ lily tepals and also explain the mechanism by which ET has opposite effects on anthocyanin synthesis in Asiatic hybrid lilies and ‘Viviana’ lilies, offering new insights into the regulatory mechanism of ERFs and providing a theoretical basis for ET derivative application in the cut flower industry.

## Results

### ET promotes anthocyanin accumulation in ‘Viviana’ lily tepals

**Figure 1:**
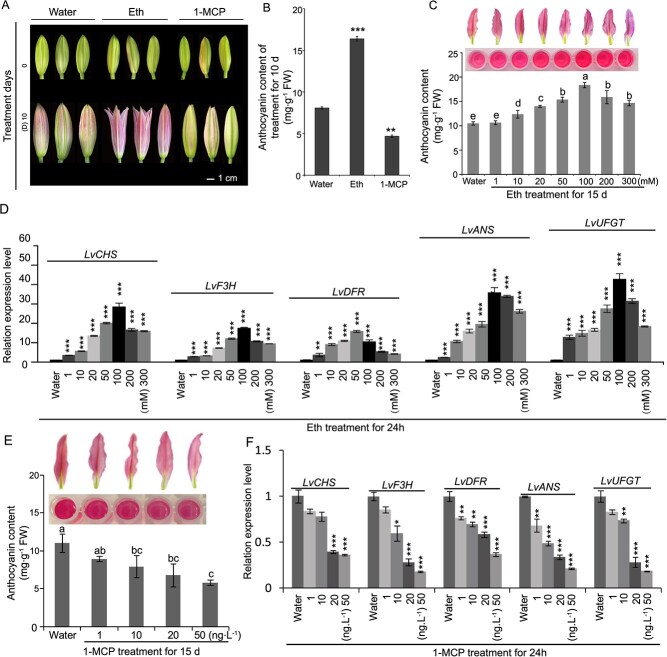
ET affects anthocyanin accumulation in ‘Viviana’ lilies. A. Coloration of flower buds at 0 and 10 days of Eth or 1-MCP treatment using water as control. Scale bar = 1 cm. B. Determination of total anthocyanin content of S2 lily buds after 10-day 100 mM Eth or 50 ng·L^−1^ 1-MCP treatment. C–F. Total anthocyanin content in S3 lily tepals after treatment with different Eth (C) and 1-MCP (E) concentrations for 15 days and structural gene expression patterns of S3 lily tepals after treatment with different Eth (D) and 1-MCP (F) concentrations for 24 h. Data correspond to the means of three biological replicates ± SD. Different letters indicate significant differences by Tukey s-b (K) test with *P* < 0.05. Asterisks indicate significant differences (Student’s *t*-test, ^*^P < 0.05, ^**^P < 0.01, ^***^P < 0.001).

To investigate whether ET influences tepal color in ‘Viviana’ lilies, flower buds were treated with Eth (100 mM) or 1-MCP (an ET inhibitor, 50 ng·L^−1^), using water as a control. After 10 days, Eth treatment significantly enhanced tepal color changes and bud maturation compared with the control, whereas 1-MCP treatment had the opposite effect (Fig. 1A). Further, Eth treatment markedly increased anthocyanin levels in flower buds compared with the control, whereas 1-MCP treatment significantly decreased them (Fig. 1B). These findings suggest that ET promotes flower maturation and regulates tepal color in ‘Viviana’ lilies.

To further explore the impact of ET on anthocyanin synthesis, we measured anthocyanin content and the expression of structural genes (*LvCHS*, *LvF3H*, *LvDFR*, *LvANS*, and *LvUFGT*) following treatment with varying Eth and 1-MCP concentrations (Fig. 1C–F). The anthocyanin content peaked at 100 mM Eth and declined at higher concentrations (Fig. 1C). Similarly, structural gene expression was highest at 100 mM Eth treatment, with a slight decrease after 24 h of treatment at 200 and 300 mM (Fig. 1D). These results indicate that ET enhances anthocyanin accumulation in lily tepals by regulating structural gene expression. In contrast, increasing 1-MCP concentrations gradually decreased the anthocyanin content (Fig. 1E) and strongly inhibited structural gene expression (Fig. 1F). These findings demonstrate that 1-MCP suppresses anthocyanin accumulation by downregulating structural gene expression.

### Screening of ERFs mediating ET regulation of anthocyanin synthesis

**Figure 2:**
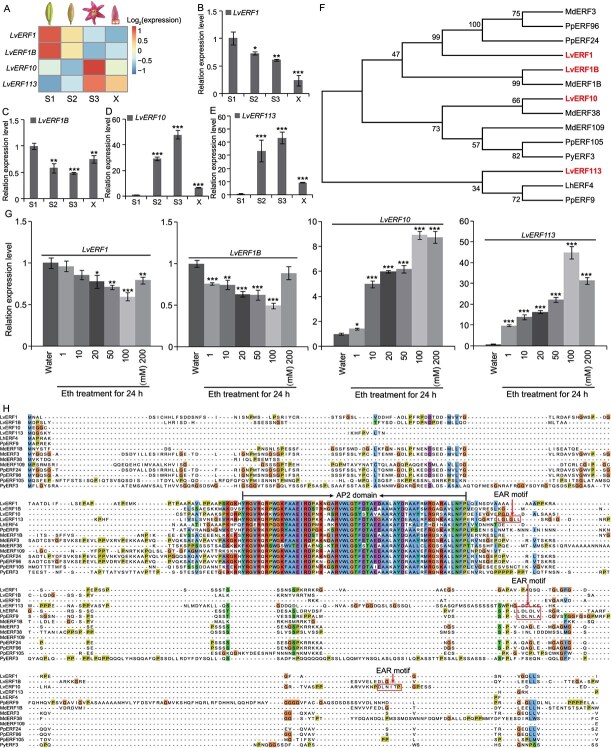
Screening of ERFs mediating ET regulation of anthocyanin synthesis. A. Differential transcripts of four screened ERFs (*LvERF1*, *LvERF1B*, *LvERF10*, and *LvERF113*) at S1–S3, determined by the FPKM values of transcriptomic data. B–E. Expression patterns of the four screened ERFs at S1–S3 and the X region of ‘Viviana’ tepals. F. Phylogenetic analysis of the candidate ERFs and selected anthocyanin synthesis-related ERFs. G. Expression changes of the four ERFs in lily tepals after treatment with different Eth concentrations for 24 h. H. Alignment of the full-length amino acid sequence of four screened ERFs and selected anthocyanin synthesis-related ERFs. Black lines indicate the conserved AP2 domain and a red frame, the EAR motifs. Data correspond to the means of three biological replicates ± SD. Asterisks indicate significant differences (Student’s *t*-test, ^*^P < 0.05, ^**^P < 0.01, ^***^P < 0.001).

To identify the ERFs involved in ET-mediated regulation of anthocyanin synthesis, we analyzed transcriptomic data from the flower developmental stages S1–S3 of ‘Viviana’ lilies (PRJNA649743), resulting in the selection of eight ERFs with significant expression changes. Among these, four *ERFs* (*LvERF1*, GenBank ID: PP586165; *LvERF1B*, GenBank ID: PP586166; *LvERF10*, GenBank ID: PP586164; *LvERF113*, GenBank ID: PP146521) were cloned for further analysis (Fig. 2A). Quantitative real-time PCR (qRT-PCR) confirmed that their expression patterns matched the transcriptome data (Fig. 2B–E). During flower development, *LvERF1* and *LvERF1B* were significantly downregulated (Fig. 2B and C), whereas *LvERF10* and *LvERF113* were upregulated (Fig. 2D and E).

Phylogenetic analysis grouped the 14 ERFs, including the 4 candidates and 10 previously identified anthocyanin-related ERFs, into two major clades. LvERF113 clustered with LhERF4 and PpERF9, known inhibitors of anthocyanin synthesis, and the remaining 11 ERFs were clustered into the other class (Fig. 2F). Our qRT-PCR analysis of Eth-treated flowers revealed distinct expression patterns: *LvERF1* and *LvERF1B* were downregulated, whereas *LvERF10* and *LvERF113* were upregulated (Fig. 2G). Sequence alignment highlighted conserved AP2 domains in all proteins and EAR motifs in LvERF10, LvERF113, LhERF4, and PpERF9 (Fig. 2H). Notably, LvERF10 contained a unique DLNxxP-type EAR motif, whereas the others had an LxLxL-type motif. Based on phylogenetic grouping and sequence features, LvERF113 is likely a potential transcriptional repressor while LvERF10 may act as an activator.

### LvMYB1 and LvMYB5 mediate ET signaling and coregulate anthocyanin synthesis

**Figure 3:**
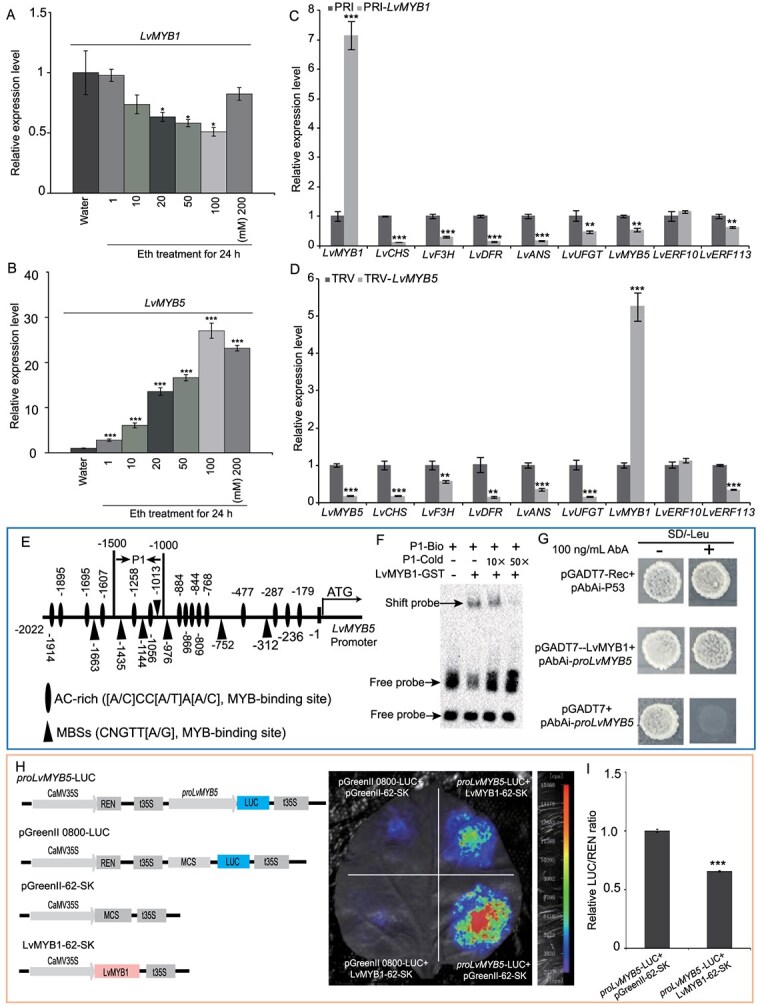
LvMYB1 and LvMYB5 mediate ET signaling and coregulate anthocyanin synthesis in ‘Viviana’ lilies. A–B. Expression patterns of *LvMYB1* (A) and *LvMYB5* (B) in lily tepals after treatment with different Eth concentrations for 24 h. C–D. Changes in the expression of structural and regulatory genes in lily tepals injected with *PRI-LvMYB1* (C), *TRV-LvMYB5* (D), and empty vector. E. AC-rich and MBSs motif analysis of the *LvMYB5* promoter and position of the EMSA probes. F–I. EMSA (F), Y1H (G), LUC (H), and LUC/REN ratio (I) assays of LvMYB1 binding to the *LvMYB5* promoter. Data correspond to the means of three biological replicates ± SD. Asterisks indicate significant differences (Student’s *t*-test, ^*^P < 0.05, ^**^P < 0.01, ^***^P < 0.001).

The role of LvMYB1 and LvMYB5 in ET-mediated anthocyanin synthesis was investigated by assessing their expression under different Eth concentrations. Our qRT-PCR analysis revealed that *LvMYB1* expression decreased significantly with 20–100 mM Eth treatment, whereas *LvMYB5* expression was significantly upregulated (Fig. 3A and B). Further, *LvMYB1* overexpression inhibited the expression of *LvMYB5*, *LvERF113*, and structural genes (Fig. 3C). Conversely, the suppression of *LvMYB5* downregulated the structural genes and *LvERF113* while promoting *LvMYB1* transcription (Fig. 3D). These findings suggest a mutually inhibitory relationship between LvMYB1 and LvMYB5 in regulating anthocyanin synthesis. Notably, neither *LvMYB1* overexpression nor *LvMYB5* suppression affected *LvERF10* expression, suggesting no regulatory relationship between *LvERF10* and the two MYBs.

*Cis*-element analysis of the *LvMYB5* promoter identified 15 AC-rich sites and seven MBS sites (Fig. 3E), indicating potential regulation by LvMYB1. Moreover, an electrophoretic mobility shift assay (EMSA) confirmed that LvMYB1 binds to the P1 region of the *LvMYB5* promoter (Fig. 3F), a finding supported by yeast one-hybrid (Y1H) and dual-luciferase reporter (LUC) assays (Fig. 3G–I). However, LvMYB5 did not directly regulate *LvMYB1* expression (Fig. S1G), suggesting the involvement of additional transcription factors (TFs) in the negative regulation of *LvMYB1* by LvMYB5.

Regarding ERFs, *LvERF10* expression was unaffected by LvMYB1 and LvMYB5, while *LvERF113* expression was inhibited by LvMYB1 and induced by LvMYB5. These results indicate that LvERF113, together with LvMYB1 and LvMYB5, plays a critical role in the regulation of anthocyanin synthesis.

### LvERF113 regulates anthocyanin accumulation in ‘Viviana’ lilies

**Figure 4:**
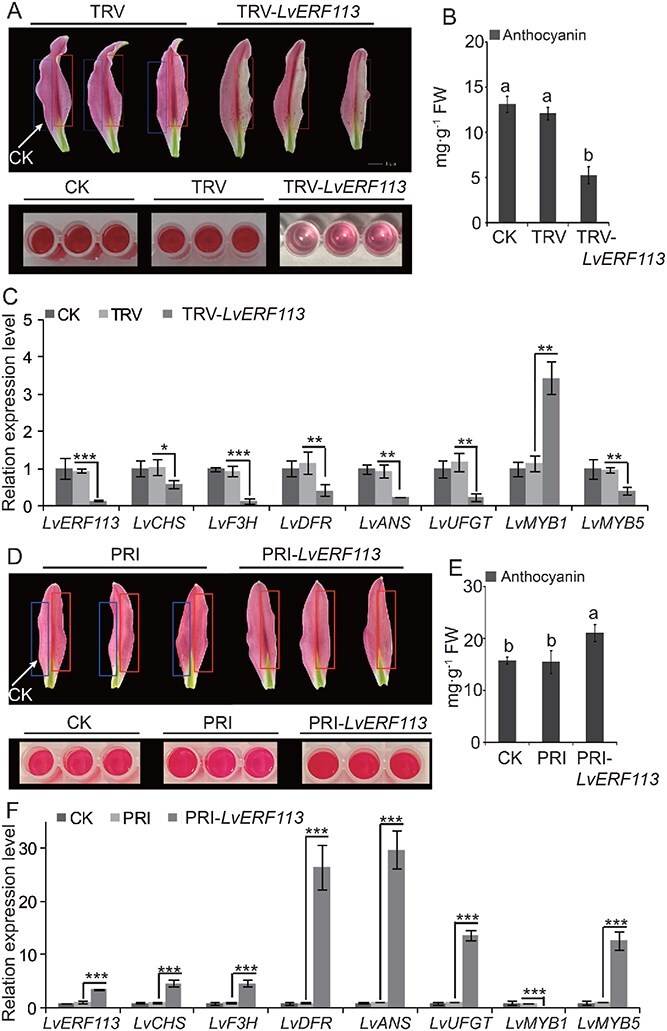
LvERF113 regulates anthocyanin accumulation in ‘Viviana’ lilies. A. Tepal phenotypes after injection with *TRV* or *TRV-LvERF113* vectors. The red box on the right side indicates the injected area and blue box on the left shows the control. B. Tepal anthocyanin concentrations after injecting the empty vector, *TRV-LvERF113*, and noninjection control. C. Expression of structural and regulatory genes in lily tepals injected with the empty vector, *TRV-LvERF113*, and noninjection control. D. Tepal phenotypes after injection with the *PRI* and *PRI101-LvERF113* vectors. E. Anthocyanin concentrations of tepals after injection with the empty vector, *PRI101-LvERF113*, and noninjection control. F. Expression of structural and regulatory genes in lily tepals injected with the empty vector, *PRI101-LvERF113*, and noninjection control. Data correspond to the means of three biological replicates ± SD. Different letters indicate significant differences by Tukey s-b (K) test with *P* < 0.05. Asterisks indicate significant differences (Student’s *t*-test, ^*^P < 0.05, ^**^P < 0.01, ^***^P < 0.001).

To examine the role of LvERF113 in anthocyanin regulation, a 200-bp fragment lacking the AP2 domain was cloned into the TRV2 vector for gene silencing. In addition, the full-length sequence was inserted into the *PRI101ON* vector for transient overexpression. Virus-induced gene silencing experiments showed that tepals injected with the *TRV* empty vector retained normal coloration (right side, red box; Fig. 4A), whereas tepals injected with the *TRV-LvERF113* recombinant exhibited lighter coloration and lower anthocyanin levels (Fig. 4A and B). The expression of structural genes and *LvMYB5* was also downregulated after *LvERF113* silencing (Fig. 4C). Conversely, transient *LvERF113* overexpression enhanced anthocyanin accumulation and upregulated the expression of structural genes and *LvMYB5* (Fig. 4D–F). Contrary to *LvMYB5*, LvERF113 inhibited *LvMYB1* expression (Fig. 4C and F). These results indicate that LvERF113 regulates anthocyanin synthesis by influencing the transcription of the structural genes LvMYB5 and *LvMYB1*.

### LvMYB5 positively regulates *LvERF113* expression, whereas LvERF113 inhibits *LvMYB1* transcription

**Figure 5:**
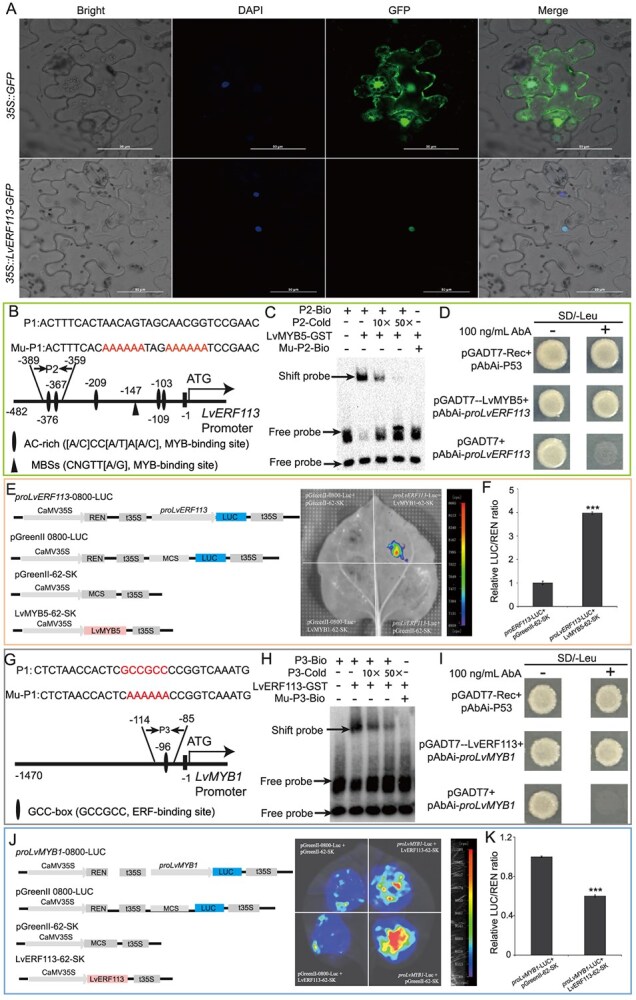
Analysis of the regulatory relationship between LvMYB5, LvMYB1, and LvERF113. A. Subcellular localization of LvERF113. DAPI nuclear staining using GFP as a control. B. *Cis*-element analysis of the *LvERF113* promoter and design of EMSA mutation probes. C–F. EMSA (C), Y1H (D), LUC (E), and LUC/REN ratio (F) assay of LvMYB5 binding to the *LvERF113* promoter. G. GCC-box analysis of the *LvMYB1* promoter and design of EMSA mutation probes. H–K. EMSA (H), Y1H (I), LUC (J), and LUC/REN ratio (K) assay of LvERF113 binding to the GCC-box in the *LvMYB1* promoter. Data correspond to the means of three biological replicates ± SD. Asterisks indicate significant differences (Student’s *t*-test, ^***^P < 0.001).

The subcellular localization assay revealed that our *LvERF113-GFP* fusion protein was located in the nucleus, confirming its function as a nuclear TF (Fig. 5A). Using genome walking, a 482-bp fragment of the *LvERF113* promoter was obtained. *Cis*-element analysis identified five AC-rich sites (−103, −147, −209, −367, and −376 bp) and one MBS site (−109 bp) within the promoter (Fig. 5B). Further, EMSA, Y1H, and LUC assays demonstrated that LvMYB5 binds to the *LvERF113* promoter, inducing its expression (Fig. 5C–F).

Analysis of the 1470-bp *LvMYB1* promoter revealed a GCC-box at −96 bp (Fig. 5G). The EMSA analysis confirmed that LvERF113-GST binds to this GCC-box upstream of the *LvMYB1* start codon (Fig. 5G). The binding strength decreased with increasing amounts of cold probes, and no binding was detected when using a mutated probe (Fig. 5H). Further assays (EMSA, Y1H, and LUC) confirmed that LvERF113 suppresses *LvMYB1* transcription by binding to its promoter (Fig. 5I–K). Collectively, these findings suggest that LvMYB5 activates *LvERF113* expression, which, in turn, inhibits *LvMYB1* transcription.

### LvERF113 promotes anthocyanin biosynthesis through its interaction with LvMYB1

**Figure 6:**
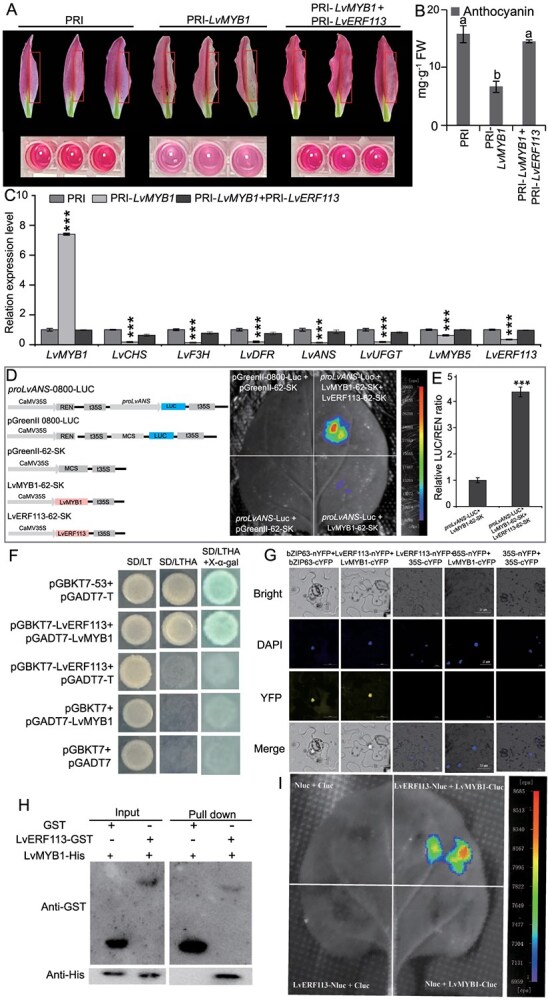
LvERF113 promotes anthocyanin biosynthesis by interacting with LvMYB1. A. Tepal phenotypes after injection of the empty vector, *PRI101ON-LvMYB1*, and coinjection of the *PRI101ON-LvMYB1* and *PRI101ON-LvERF113* vectors. The red box on the right indicates the injected area. B. Anthocyanin contents of tepals after injecting the empty vector, *PRI101ON-LvMYB1*, and coinjection with *PRI101ON-LvMYB1* and *PRI101ON-LvERF113*. C. Changes in expression of *LvMYB1* and structural genes in tepals injected with the empty vector PRI101ON*N-LvMYB* or coinjected with *PRI101ON-LvMYB1* and *PRI101ON-LvERF113*. D–E. The LUC (D) and LUC/REN ratio (E) assay of LvMYB5 binding to the *LvANS* promoter. F–I. Y2H (F), BiFC (G), pull-down (H), and Firefly luciferase complementation imaging assay (I) for LvERF113 and LvMYB1. Data correspond to the means of three biological replicates ± SD. Different letters indicate significant differences by Tukey s-b (K) test with *P* < 0.05. Asterisks indicate significant differences (Student’s *t*-test, ^***^P < 0.001).

Transient *LvMYB1* overexpression in lily tepals significantly reduced anthocyanin levels compared with the control (Fig. 6A and B) and inhibited the expression of the structural genes (*LvCHS*, *LvF3H*, *LvDFR*, *LvANS*, and *LvUFGT*; Fig. 6C). Interestingly, in a cotransformation assay with LvERF113, the inhibitory effect of LvMYB1 on anthocyanin synthesis was mitigated (Fig. 6A–E). Although LvERF113 could not suppress *LvMYB1* transcription due to its overexpression under the 35S promoter, it effectively neutralized LvMYB1’s inhibitory function, suggesting a potential interaction at the protein level between LvERF113 and LvMYB1.

To confirm this interaction, we performed yeast two-hybrid (Y2H), bimolecular fluorescence complementation (BiFC), pull-down, and Firefly luciferase complementation imaging assays (Fig. 6F–I). These experiments demonstrated that LvERF113 physically interacts with LvMYB1. Thus, LvERF113 not only suppresses *LvMYB1* transcription but also counteracts its inhibitory activity at the protein level, promoting anthocyanin synthesis.

## Discussion

ET plays a crucial role in flower development and fruit ripening [42–44]; that is why its derivatives, Eth and preservatives, are widely utilized in agricultural practices. This study demonstrated that ET promotes anthocyanin synthesis in ‘Viviana’ lily tepals through Eth and 1-MCP treatments (Fig. 1A). This result differs from previous research on the effect of ET on anthocyanin synthesis in Asian hybrid lilies [40, 41]. Although the role of ET in regulating flowering time has been extensively researched [45, 46], its participation in anthocyanin synthesis in lilies remains relatively underexplored. Understanding the regulatory pathways through which ET influences anthocyanin synthesis provides valuable insights for lily breeding and optimizing the application of ET derivatives in the floral industry.

Anthocyanins play a role in plant development and stress tolerance, with their accumulation being regulated to ensure appropriate spatiotemporal expression [4]. Previous research identified LvMYB1 and LvMBY5 as key regulators of anthocyanin synthesis regulation in ‘Viviana’ lilies [39]. We confirmed that they belong to the SG4 and SG6 subgroups of R2R3-MYB, respectively (Fig. S1A), and that they respond to ET signals (Fig. 3A and B). Our functional assays revealed that LvMYB1 negatively regulated anthocyanin synthesis, whereas LvMYB5 acted as a positive regulator (Fig. S1E and F). These findings suggest that LvMBY5 functions as part of the MBW complex, mediating ET-mediated regulation of anthocyanin synthesis together with LvMYB1.

**Figure 7:**
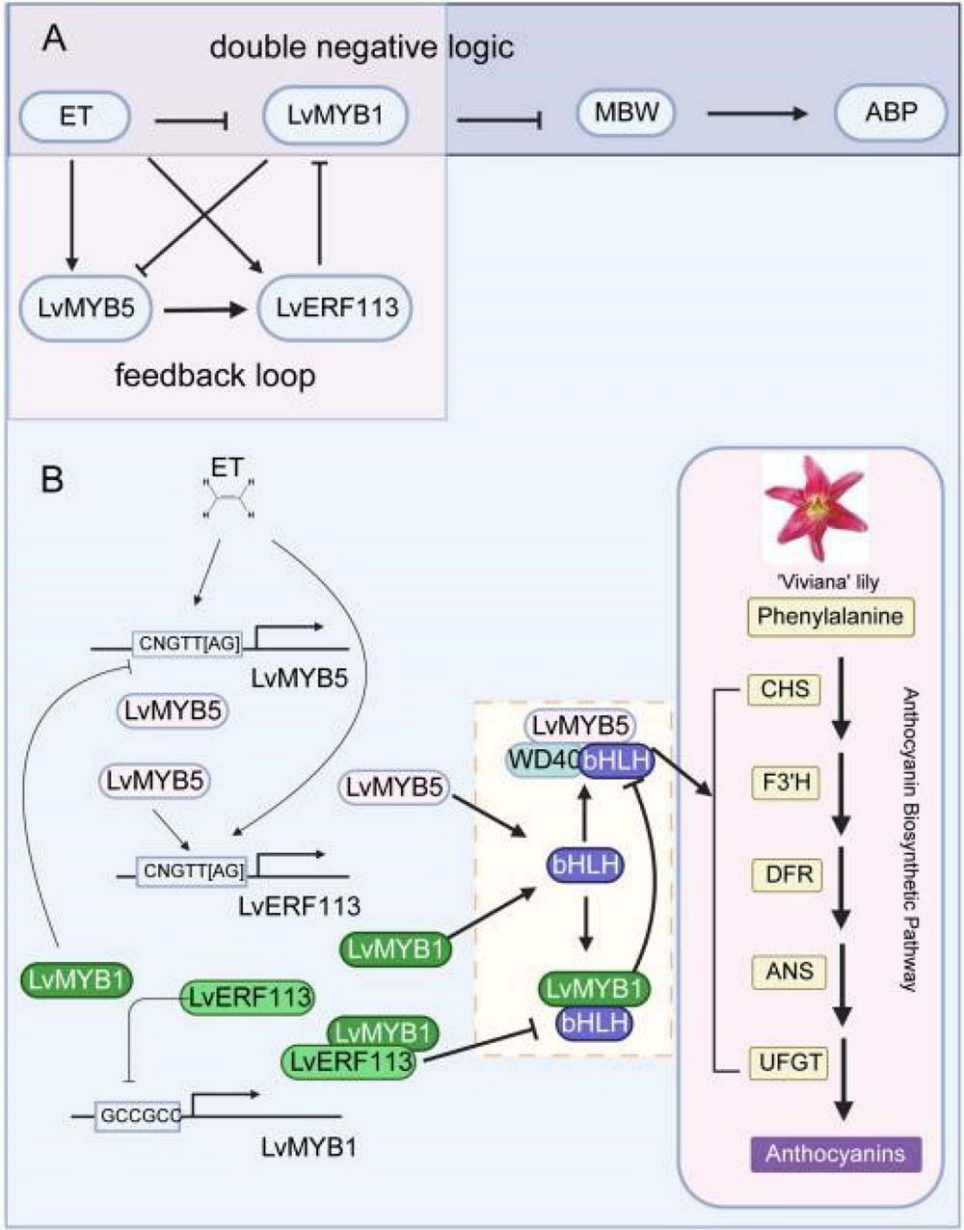
Model of ET-regulated anthocyanin biosynthesis via the LvMYB5-LvERF113-LvMYB1 module in ‘Viviana’ lilies. Dashed boxes represent the regulatory links speculated based on the literature and the thick line indicates the regulatory relationship identified between the protein and the protein complex.

Studies on MYB inhibitors in other species suggest a ‘double-negative logic’ mechanism, and they often form feedback inhibition loops with MYB activators to regulate anthocyanin synthesis [4, 18, 47]. Our results are in alignment with this mechanism; in fact, during ET signal transmission, LvMYB1 is inhibited by ET following the ‘double negative logic’ (Fig. 7A). However, the regulatory relationship between LvMYB1 and LvMYB5 deviates from the ‘feedback inhibition loop’ (Fig. 3C and D). Instead of a feedback inhibition loop, we identified a mutually inhibitory relationship wherein LvMYB1 suppresses *LvMYB5* transcription directly, but LvMYB5 does not directly inhibit *LvMYB1* (Figs 3F–I and S1G). This suggests the presence of an intermediary factor mediating *LvMYB1* suppression by LvMYB5.

LvERF113, an ERF containing an EAR motif, mediates the ET-regulated pathway of anthocyanin synthesis (Figs 2G and  4). Phylogenetic analysis identified LvERF113 as homologous to LhERF4 and PpERF9 (Fig. 2F and H), known inhibitors of anthocyanin synthesis in Asiatic hybrid lilies and ‘Hongzaosu’ pears, respectively [28, 40]. However, unlike its homologs, LvERF113 enhances anthocyanin accumulation in ‘Viviana’ lilies. *LvERF113* silencing decreased the anthocyanin content and structural gene expression, while overexpression significantly increased both (Fig. 4).

In Asiatic hybrid lilies, both LhERF4 and LhERF061 mediate the negative ET regulation on anthocyanin synthesis and can inhibit anthocyanin synthesis by suppressing *LhMYBSPLATER* expression [40, 41]. In ‘Viviana’ lilies, LvERF113 promotes anthocyanin synthesis by inhibiting *LvMYB1* transcription and enhancing *LvMYB5* expression (Fig. 4C and F). The *LvMYB1* promoter contains a GCC-box, allowing direct regulation by LvERF113 (Fig. 5G–K), whereas the *LvMYB5* promoter lacks such motifs (Supplementary Data 3), suggesting indirect regulation. LvMYB5 positively regulates *LvERF113* transcription by binding to its promoter, forming a feedback loop within the LvMYB5-LvERF113-LvMYB1 module (Fig. 7A). In this module, ET enhanced *LvERF113* and *LvMYB5* expression, suppressing *LvMYB1* and alleviating its repressive effects on structural genes (Fig. 7B). The module is a branch of the anthocyanin synthesis regulatory network that mediates ET signals. It regulates anthocyanin synthesis by influencing the expression and function of LvMYB5 in the MBW complex.

LvERF113 employs a dual mechanism to neutralize LvMYB1’s inhibitory function. First, it suppresses *LvMYB1* transcription by binding to its promoter. Second, it interacts with LvMYB1 so that it loses the ability to bind to structural gene promoters (active negative regulation) (Fig. 6D–E) or interact with bHLH proteins (passive negative regulation). This dual mechanism has parallels in other species, such as the MdERF1B-MdMYB9/11 interaction in apples [48] and the PyERF3-PyMYB114 interaction in pears [49]. In Asiatic hybrid lilies, LhERF061 can inhibit the positive regulatory function of LhMYBPSLATTER at the posttranslational level by interacting with LhMYBPSLATTER [41]. We further validated the LvERF113-LvMYB1 interaction using Y2H, BiFC, pull-down, and Firefly luciferase complementation assays in ‘Viviana’ lilies (Fig. 6F–I). This indicates that LvERF113 not only regulates *LvMYB1* expression but also affects protein function at the post-translational level, and explains why ET inhibits anthocyanin synthesis in Asian hybrid lilies but promotes it in ‘Viviana’ lilies. The regulatory roles of ERFs and MYBs in ET-mediated anthocyanin synthesis differ across plant species due to variations in the combinations of TFs. The coregulation of anthocyanin synthesis by MYBs and ERFs has demonstrated that the coactivation between activators promotes anthocyanin synthesis [34, 48, 50], and the coregulation between activators and inhibitors inhibits anthocyanin synthesis [27, 28]. Unlike previous studies, in ‘Viviana’ lilies, LvERF113 operates by inhibiting the inhibitor LvMYB1, thereby promoting anthocyanin accumulation. This novel dual inhibitory mechanism broadens our understanding of ERF-MYB coregulation in anthocyanin synthesis.

In summary, LvERF113, a negative ERF, positively regulates anthocyanin synthesis in ‘Viviana’ lilies through the LvMYB5-LvERF113-LvMYB1 module. ET upregulates *LvERF113* and *LvMYB5* transcription, which together suppress *LvMYB1*, alleviating its repressive effects on structural genes and promoting anthocyanin biosynthesis. This study not only uncovered a novel ET-mediated regulatory pathway in lilies but also provided valuable insights for improving anthocyanin-related traits in horticulture. In addition, the present findings provide a theoretical foundation for the application of ET derivatives in the cut flower industry.

## Materials and methods

### Plant materials and growth conditions

The experimental materials, ‘Viviana’ (*Lilium hybrid cultivar*) and tobacco (*Nicotiana benthamiana*), were grown in a greenhouse. The temperature was consistently maintained at 25°C ± 2°C, with a light cycle consisting of 16/8 h. The relative humidity in the environment was kept at 60%. Lilies were grown for about 20 days to initially form flower buds and begin sampling. The tepals were divided into three stages: the bud stage (S1, occurs 20 days after bud creation), the bud coloring stage, (S2, 30 days after bud formation), and the peak blooming stage (S3, 40 days after bud formation). The X region was the uncolored area at the base of the S3 tepals. Three biological replicates of tepal samples were collected from each developmental period. Each replicate was taken from a mixture of three samples of the ‘Viviana’. Instantly preserve the gathered samples by subjecting them to rapid freezing in liquid nitrogen and storing them in an ultra-low temperature refrigerator. *N. benthamiana* seedlings, aged 4–6 weeks, were used in the experiments.

### Eth and 1-MCP treatment

To clarify the effect of ET on lily flower coloring, S1 stage lily buds with good growth conditions were selected and sprayed with water (CK) and 100 mM Eth every day for 10 days. We observed the coloring of flower buds under water and Eth treatment groups at 10 days, and took photos to record. We extracted anthocyanins from tepals on the 10th day after treatment. To determine the impacts of various concentrations of Eth and 1-MCP on lily flower coloring, the following concentrations were selected for Eth and 1-MCP treatments: Eth (1, 10, 20, 50, 100, 200, and 300 mM) and 1-MCP (1, 10, 20, and 50 ng·L^−1^). The S1 and S3 stage lily buds treated with Eth and 1-MCP were bagged and kept for 24 h. After removing the bags, S3 materials were used for the qRT-PCR analysis and S1 materials were cultured normally for 15 days to measure the anthocyanin content.

### Total anthocyanins extraction and determination

The bloom ‘Viviana’ tepals treated with Eth and 1-MCP were used to extract total anthocyanins with three biological replicates. The assay procedure was delineated in the following manner: The fresh sample (0.1 g) was pulverized into powder using liquid nitrogen. The 600-μl extraction solution, which consisted of a methanol solution containing 1% HCl, was added to the mixture and incubated at 4°C overnight in the dark. After the total anthocyanins were fully extracted, 400-μl chloroform and 400-μl water were added to remove the chlorophyll. The plant materials were precipitated by centrifugation at 10 000*g* for 5 min at 4°C. We aspirated the supernatant and determined the absorbance values at 530 nm (A530) and 657 nm (A657). The total anthocyanin content was calculated using the formula: (A530 − 0.33 * A657) g^−1^ fresh weight [51].

### Total RNA isolation, cDNA synthesis, and DNA extraction

The EASYspin Plus spin kit (Aidlab, Beijing, China) was utilized for the extraction of total RNA. Using MonScript Synthesized First Strand cDNATM RTII coupled with dsDNase (Monad, Suzhou, China), cDNA synthesis was carried out. The Modified CTAB Plant DNA Kit (Aidlab) was used to extract the DNA.

### qRT-PCR analysis

The MonAmpTM ChemoHS qPCR-Mix (Monad, Suzhou, China) was used for qRT-PCR. Triplicates of each reaction were carried out to analyze the gene expression in the ‘Viviana’ tepals. The reaction system was as follows: 5 μl of MonAmpTM ChemoHS qPCR-Mix, 0.3 μl of gene-specific primers, 1 μl of 10-fold diluted first-strand cDNA, and 3.4 μl of deionized water, and the amplification procedure was as follows: 95°C for 5 min, and 40 times of 95°C (10 s) and 60°C (15 s). The actin gene was used as a reference.

### Bioinformatics analysis

The Jalview was used for the alignment of MYBs and ERFs [52]. The phylogenetic tree was built using MEGA v7.0 software following parameters: the NJ method + JTT + G, 50% site coverage cutoff, and 1000 bootstrap replications [53]. The online software, Multiple Em for Motif Elicitation 5.4 (MEME), was used to calculate the conserved motifs and can be accessed at https://meme-suite.org/meme/tools/meme.

### Promoter cloning and binding site analysis

Promoter cloning was performed using the Genome Walking Kit (Takara, Beijing, China). The 500 ng of lily DNA was used for the first step of the reaction, and a 100-fold dilution of the PCR product was used as a template for the second- and third-step reactions. The binding sites on the promoters of *LvMYB1*, *LvMYB5*, and *LvERF113* were searched based on the conserved motifs (GCC-box, AC-rich, and MBSs).

### VIGS analysis

The tobacco rattle virus (*TRV*) VIGS vector was used to silence the *LvMYB5* and the *LvERF113* genes. Specific primers (Supplementary Data 1) with EcoRI and BamHI recognition sites were used to amplify 300-bp *LvMYB5* and 200-bp *LvERF113* fragments from ‘Viviana’ cDNA. The target fragments were inserted into the *TRV2* vector using infusion ligase and then transformed into DH5α cells and the positive clones for DNA sequencing. The verified plasmid was introduced into *GV3101* competent cells using freeze–thaw. *Agrobacterium* colonies (*TRV1, TRV2, TRV-LvMYB5, TRV-LvERF113*) were cultured in liquid YEP medium (50 mg·L^−1^ kanamycin and rifampicin) at 28°C. The cultures were incubated with agitation until OD_600_ reached 0.8. Bacteria were centrifuged at 4°C, 5000 rpm, for 10 min to precipitate the bacterial bodies. The cells were washed three times with 10-mL resuspension solution (10 mM·mL^−1^ MES, 10 mM·mL^−1^ MgCl_2_, 20 μM·mL^−1^ AS, pH 5.6), resuspended, and placed in the dark at 28°C for 3 h. The cells of *TRV2* and *TRV-LvMYB5*, *TRV-LvERF113* were mixed with *TRV1*, respectively, then the mixed cells were injected into the slightly colored outer tepal epidermis of S2 stage ‘Viviana’ until the injected area reached more than 50% of the tepal area. Three biological replicates of each treatment were performed. The injected plant materials were placed in darkness for 24 h and 16/8-h light cycle for 7 days. After this, these were photographed and collected for further analysis.

### Transient overexpression and coexpression assays

The *PRI101ON* vector was used to overexpress the genes of *LvERF113* and *LvMYB1*. The *LvERF113*-CDS and the *LvMYB1*-CDS were amplified from the ‘Viviana’ tepals, and the *CaMV35S* promoter was integrated into the *PRI101ON* vector by homologous recombination. The method of transforming the recombinant vector into *DH5α* and *GV3101*, treatment of the organisms, and injection method were as described in VIGS. Meanwhile, in the coexpression assays, the recombinant vectors of *PRI101-LvERF113* and *PRI101-LvMYB1* were mixed at 1:1 and injected into lily tepals using the same method as VIGS.

### Subcellular localization

To clarify the distribution of LvERF113 protein in cells, the *pCAMBIA1300-LvERF113-GFP* recombinant vector was constructed by homologous recombination for subcellular localization analysis. *Agrobacterium tumefaciens GV3101* containing the *pCAMBIA1300-LvERF113-GFP* recombinant vector and *pCAMBIA1300-GFP* (control) were injected into the tobacco leaves, respectively. The GFP fusion proteins and the positions of the nuclei using a laser confocal microscope after 72 h processing.

### EMSA assay

The specific binding ability of LvMYB1 to the *LvMYB5* promoter, LvMYB5 to the *LvERF113* promoter, and LvERF113 to the *LvMYB1* promoter were verified using the purified LvMYB1-GST, LvMYB5-GST, and LvERF113-GST fusion proteins. The biotin probes (P1-Bio, P2-Bio, and P3-Bio), cold probes (P1-Cold, P2-Cold, P3-Cold), and mutated probes (Mu-P2-Bio, Mu-P3-Bio) of the three sites were synthesized or amplified (Figs 4C and 6B and F). The binding ability was verified by competition with the probes of Cold (10× and 50×) and Mu-Bio. EMSA assay was performed using a chemiluminescent EMSA kit (Beyotime, Shanghai, China).

### Y1H analysis

The 630-bp *LvMYB1*-CDS, the 720-bp *LvMYB5*-CDS, and the 603-bp *LvERF113*-CDS fragment were transformed into the *pGADT7* vector to construct the *pGADT7-LvMYB1*, *pGADT7-LvMYB5*, and *pGADT7-LvERF113* vectors by homologous recombination, respectively. The recombinant vector of *pAbAi-proLvMYB5*, *pAbAi-proLvERF113*, and *pAbAi-proLvMYB1* were constructed by analyzing the MYB (AC-rich and MBS) and ERF (GCC-box) binding sites and amplifying promoters of the *LvMYB5*, *LvERF113*, and *LvMYB1.* The pAbAi recombinant vector was linearized using restriction endonuclease *Bstb I* and transferred into *Saccharomyces cerevisiae* Y1H Gold competent cells. We incubated it on SD/-Ura plates at 30°C for 3–4 days to screen the positive colonies. Receptor cells containing pAbAi recombinant vector were prepared by inoculating the well-grown positive strains into the YPDA medium for expansion. The *pGADT7-LvMYB1*, *pGADT7-LvMYB5*, and *pGADT7-LvERF113* were transfected into the *pAbAi-proLvMYB5*, *pAbAi-proLvERF113* and the *pAbAi-proLvMYB1* competent cells, respectively, and incubated on SD/−Leu plates at 30°C for 3–4 days. We picked up the positive colonies and placed them into 20 μl of 0.9% NaCl solution. We dropped 8 μl of the solution onto the SD/−Leu plates with or without 100 ng·mL^−1^ aureobasidin A (AbA), respectively, and incubated it at 30°C for 3–4 days. Meanwhile, the yeast cells cotransformed with *pGADT7-Rec-P53* and *pAbAi-P53* were used as the positive control.

### LUC analysis

The promoters of 2022-bp *LvMYB5*, 482-bp *LvERF113*, and 1470-bp *LvMYB1* were ligated into the *pGreenII-0800-Luc* vector using homologous recombination to construct the *proLvMYB5-Luc*, the *proLvERF113-Luc*, and the *proLvMYB1-Luc* recombinant vectors. The CDS of *LvMYB1*, *LvMYB5*, and *LvERF113* were ligated into the *pGreenII-62-SK* to construct *LvMYB1-62-SK*, *LvMYB5-62-SK*, and *LvERF113-62-SK* recombinant vectors. All the specific primer sequences used are summarized in Supplementary Data 1. The *pGreenII-0800-Luc* and *pGreenII-62-SK* empty vectors were used as the effector. The *proLvMYB5-Luc* and *LvMYB1–62-SK*, the *proLvERF113-Luc* and *LvMYB5–62-SK*, the *proLvERF113-Luc* and *pGreenII-62-SK*, the *pGreenII-0800-Luc* and *LvMYB5–62-SK*, and the *pGreenII-0800-Luc* and *pGreenII-62-SK* were mixed 1:1 and incubated in the dark at 28°C for 3 h, then injected into tobacco leaves. After 24 h of dark treatment, d-luciferin potassium salt was evenly applied to the injected area. After treatment in the dark for 5 min, the fluorescence signal intensity of tobacco leaves was observed using an *in vivo* fluorescence imaging system. The same analytical method was used to detect the others. The activity ratio of firefly luciferase and *Renilla* luciferase was tested using a dual-luciferase reporter assay system (E1910, Promega, USA) following the manufacturer’s instructions, The experiment was performed with three biological replicates, and each biological replicate included three technical replicates.

### Y2H analysis

To verify the interaction between LvMYB1 and LvERF113 proteins, specific primers containing EcoRI and PstI restriction endonuclease recognition sites were used to amplify a 603-bp CDS of *LvERF113* from ‘Viviana’ cDNA. A 630-bp CDS of *LvMYB1* was amplified from ‘Viviana’ cDNA using specific primers containing EcoRI and BamHI restriction endonuclease recognition sites. The *pGBKT7-LvERF113* and the *pGADT7-LvMYB1* recombinant vectors were constructed using infusion ligase, and all the specific primer sequences used are summarized in Supplementary Data 1. We transformed the recombinant vector into Y2H Gold yeast receptive cells and cultured them on SD/− Trp/− Leu plates for 3–4 days at 30°C. Then, the yeast was transferred to the plates containing SD/−Trp/−Leu/-His/−Ade medium containing X-α-Gal. Meanwhile, yeast cells cotransformed with *pGBKT7–53* and pGADT7 were added as a positive control.

### BiFC analysis

To further verify the interactions between LvMYB1 and LvERF113 proteins, specific primers containing SacI and SalI restriction endonuclease recognition sites were used to amplify a 603-bp CDS of *LvERF113* and a 630-bp CDS of *LvMYB1.* The *pSPYNE-LvERF113*-*nYFP* and the *pSPYCE-LvMYB1*-*cYFP* recombinant vector were constructed using infusion ligase. All the specific primer sequences used are summarized in Supplementary Data 1. All the recombinant vectors were transformed into *GV3101* receptor cells with the *pSPYNE-bZIP63-nYFP* and *pSPYCE-bZIP63-cYFP* as positive control and *35S-nYFP* and *35S-cYFP* as negative. We adjusted the OD_600_ of the bacterial solution to 0.8, and mixed the *pSPYNE-LvERF113-nYFP* and the *pSPYCE-LvMYB1-cYFP* with a 1:1 ratio, then incubated them in the dark at 28°C for 3 h. The *pSPYNE-LvERF113-nYFP, 35S-cYFP*, *35S-nYFP*, and the *pSPYCE-LvMYB1-cYFP* were prepared and injected into tobacco leaves in the same way. After 48 h, we observed the signals and nucleus location of the YFP fusion protein using laser confocal microscopy.

### GST-pull down

The recombinant proteins were purified using a GST-tagged protein purification kit (Beyotime, P2262) and a His-tagged protein purification kit (Beyotime, P2226). The purified GST-tagged protein and LvERF113-GST recombinant protein were mixed with LvMYB1-His recombinant protein in equal proportions, respectively, and then placed in 1 mL of binding buffer (50 mL: 349 mg Tris–HCl, 292 mg NaCl, pH = 7.5; 125 μl Triton X-100, 136 μl β-mercaptoethanol) and stand at 4°C for 2–4 h. The products were mixed with GST resin and incubated on ice for 2 h before elution. Input and pull-down were added to SDS-PAGE polyacrylamide gel electrophoresis for Western blotting using anti-GST and anti-His, respectively.

### Firefly luciferase complementation imaging assay

The specific primers containing BamHI and SalI restriction endonuclease recognition sites were used to amplify a 603-bp CDS of *LvERF113*. The *LvERF113*-*Nluc* recombinant vector was constructed using infusion ligase. A 630-bp CDS of *LvMYB1* was amplified using the specific primers containing BamHI and PstI restriction endonuclease recognition sites. The *LvMYB1*-*Cluc* recombinant vector was constructed using infusion ligase, and the *LvERF113-Nluc* and the *LvMYB1-Cluc* recombinant vectors were constructed using homologous recombination. The recombinant vectors and the negative controls *pCAMBIA1300-Nluc* and *pCAMBIA1300-Cluc* were transformed into *GV3101*. The agrobacterium mixture was configured according to the BiFC assay operation and was injected into tobacco leaves. The injected leaves were treated and photographed as described previously.

### Statistical analysis

Each experiment was performed in triplicates, and the sampling process was completely random. Software from SPSS Inc., Chicago, IL, USA, was used to analyze the bioassay data. The average of three replicates ± standard deviation includes all experimental data. The *post hoc* Tukey s-b (K) test was used to conduct the anthocyanin content analysis following the ANOVA. The letters indicate significant differences between the different values (*P* < 0.05). The qRT-PCR analysis utilized the Student’s *t*-test, with significance levels set at ^*^P ≤ 0.05, ^**^P ≤ 0.01, and ^***^P < 0.001.

## Supplementary Material

Web_Material_uhaf059

## Data Availability

RNA-Seq data in this study are openly available in the NCBI (accession number, PRJNA649743). Other relevant data are included in the manuscript and supporting information.
