# Supplementary material for: Ethylene promotes anthocyanin synthesis in ‘Viviana’ lily via the LvMYB5-LvERF113-LvMYB1 module
Source: Hortic Res. 2025 Feb 25;12(6):uhaf059. doi: 10.1093/hr/uhaf059 (PMC12023856; doi:10.1093/hr/uhaf059)
Supplement: Web_Material_uhaf059 [file web_material_uhaf059.zip › Supplementary date 3.docx]

**Supplementary data 3. LvERF113, LvMYB1 and LvMYB5 cis-acting element position on the promoter.**

**>LvMYB1**

**GCC-box:GCCGCC**

TGTCGAGAGAAAAGAACACGCAAACTGACGGAGAGACTCTCCTCTTTTTTTTTTGTCTAAACATGTTTTATCCCTTTGTTTTGCTATCTAGACTTCACGTATATCTTCTTGACTAAACAAGTTTCGATCCTACATCTATGTGCCCCACCCCTAACATCATAAATATTCTTATAAGAGTCGTCCTATTCAAACTATTTTGTTATCTAGACCTCACGTATATCTTCTCGACTAAACAAGTTTCGATCCTACATCTATGTGCCCCACTCCTAACATCATAAATATTCTTATAAGAGTCGTCCTATTCAAACTATTTTGTTATCTAGACCTCACACATATCTTCTTGACTAAACAAGTTTCGATCCTTCTGGTCGACAGAAAAGAACACGCAAACTGACGGAGAGACTCTCCTCTTTTTTTTTTTTGTCTAAACATGTTTTATCCCTTTGTCTTGCTATCTAGACTTCACGTATATCTTCTTGACTAAACAAGTTTCGATCCTACATCTATGTGCCCCACTCCTAACATCATAAATATTCTTATAAGAGTCGTCCTATTCAAACTATTTTGTTATCTAGACCTCACACATATCTTCTTGACTAAACAAGTTTCGATCCTTCTCGTCGAGTGAAAAGAACACGCAAACTGACGGAGAGACTCTCCTCTTTTTTTTTTTGTCTAAACATGTTTTATCCCTTTGTTTTGCTATCTAGACTTCACGTATATCTTCTTGACTAAACAAGTTTCGATCCTACATCTATGTGCCCCACTCCTAACATCATAAATATTCTTATAAGAGTCGTCCTATTCAAACTATTTTGTTATCTAGACCTCACACATATCTTCTTGACTAAACAAGTGATAATCTTATATCTATATACCATACACATTCTTATAAGAGTCGTCCTATTCAAATATTAATGCTTTGGTGAAGTGGTTGGCCTTCGACACTGTTTTGCTTTATTAACTTATAAGATTTTGGACTTTCACTAACTTATAAGATTTTTTTTTTTTTCTTCTTCAATGTAACACCATCTTTATATTAATATAACTGGTCCGGGTCACTGCCCAACCCGCCCCACCCTGTCGGGTCGGGGATATTAAAAAAAAACAAAAAAAAAACAAAATTGTAACTTCCCAGAACCTTGGAGGTAGGAGAGGTAAAAAGCTATTGAGGACTTATGAGCTACCATTGACAGAATTTGAATCCAATCAAATAGGACCTTGACAGCATCCAAACAAAAACGGACCAAAACTAGAACATGTAACTCTGAGGGTGTAATTCATTAAAAACCAACTGGTTAGAAAAATGGAACACCCAGGTAAAGAAAGCAGAGCTCACCTACCAACCAAATCCAGCTCTAACCACTCGCCGCCCCGGTCAAATGGGATAGAGTCAATTCCGGGTCACAACCTCCCCTCCCATAAATACCCCTCCTCCCCTCTCTCCATCCCTCCCACTCCCTCTCCACCATG

**>LvERF113**

**AC-rich :[A/C]CC[A/T]A[A/C] MBSs :CNGTT(A/G)**

AGTCGAGAGAGATGAAGAGAAAGGTCGAATTGGGTACCGCCGCCAATTTTAATCAAAGTGGGAATATTGCTGATAGTTCATTGTCCTTCACTTTCACTAACAGTAGCAACGGTCCGAACCTCATAACAACTCAAACAAATTCTCAAGCGCTTTCACAACCAATTGCCTCCTCTAACGTTCATGATAACTTCATGAATAATGAAATCACGGCTAGTAAAATTGATGATGGTAATAATTCAAAACCACTGTCACCTGGTTGGACGGACCAAACTGCGTATAACGCGTTTGGAATCACTACAGGGATGTTTAATACCACTACAATGGATCTGTTATATAACTATCTATTCGATGATGAAGATACCCCACCAAACCCAAAAAAAGAGATCTTTAATACGACTCACTATAGGGCGAGCGCCGCCATGGAGTACCCATACGACGTACCAGATTACGCTCATATGGCCATGGAGGCCAGTGAATTCATG

**>LvMYB5**

**AC-rich :[A/C]CC[A/T]A[A/C] MBSs :CNGTT(A/G)**

AAGTGTCTGGTATTCAGAATTGACAGTATGGAACTACATTGTTATGTGGATGCTAATATGGGAGGAGATCTGAATTGGCGGAAGAGTACCATTGGCTACGTGTATATATTTGGTAAAACTGCTATGAGTTGGGCTTCTAAGTTACAGAAAATAGTTACATTATCAACGACGGAAGTAGAGTACGTGGTGATCACGGAAATAAGTAAATAAATAGTATGGCTATAGAATTTTATGGAGGAACTGGGAGTGAGATACGGAAAGAGGACGTTGCATAGCGATAGCCAAAGTGTGATATATCTGGCACAGAATTCGGTGTTTCATTTGAGAACCAAACACATCCAGATCATGTACCATTTTATCAGTTATTTGCTCGAGAAAAAGGTACTACGACTAGTGAAGATTGTCGATATGAAAAACCAAACAGATATATTTACTAAGGGAGTTCCTTTGGAGAAGCTTGGGCTGTGATGATTTGAGAAAACCTATATCAGGAGCCTCTAGTTAGCTCCTATAGTTTTATTTATTTATTTTCTTTTTAGAATAAATAATTGGAGCAGATTCACATCCACATATTCAAATAAGTTTCTCAGTTACACTGGAACTTAGTATAGGACTCGTCCTGTAGTAAAGTGATTGGTTGGACACTTGTCCTTGTGGAGTCCCATAACTCCCCGTCAACCCATCGGATTGATGGAATAACAAAACAATCTTATTTTTCTCTCTGCCCACCATTTTACTGTATGGTTTCTGGATTTTTTTTTCCTTTTGGTTTAAGCAGGGCCAAAAGGCAAACATGAAGTGAGTTGGAGGTGTCCATCTCACTACACAGATCCCCATTGTAGTTGAACAAAACAAAAGGCCCACAACTTCATCTCCTTCAGTTGCTAATAGAGCCACGAGAATTAATCCCTTCTGCCTTATTCCTCTTAATTTTATTGGAGATTTCAAACACCGACGACCCACGACACCTACTGTCTTTCAGCATTTATCAGTGGTCCATCATCGTAAGCGGTTGTGGACCACCGGATGGTCATAAATATATGGATCTGTTATGATAGTATGGAATGGTTGTAGAGTAATAATAGATGAAGCTACGACTTTTTGTAATCTCTACCAGTAGTCACTAATCTAACATCAATTTGGTTATGAGAAAAATGTTGGGAGGTTGCATGAAAAATGTTGGGAAATTATCGGACATTGTAGAGAATGTTGTTTTGGGAACAAAGGTTCAATTACAAATCTTGCACCCCATTGGTTGGTGAGAGTGTGGCTGTTAGTTAGTGGGTGTCTCTTTAAATAAATTATATTATGTTCTAATAAAATGAGCGAGATTTGCTTTTAAATTTAAATTTTATTTATTGTGATATGCTACAATAATTTCCTGAAAATCGATGGTTTCATTTTTGGCGTTCAAATGATGTTGTAGTGAGTGTCTCATGCATGGGGAGGAAGGAGACCACCGATGTTATATCACATATTCTAAATTCGCTTCATGGAGCAAACTCCCTTCAAATGAAAGGAGCAAAGAAAATCTCTAAAGAAGTCTTTGGGTGAGGTTGTTTATGGTGTTGGCTTTACGGTGTTCTTCTTAGAGGAAGTAAAATGCATATATAGAGATTGGTGATGTTTTAATGGAGAGTATACAACTATTCTTAGAACTTGGTGGGAATACATCATGTATCATCTTTGACGATACAAATATAGTTCTACCCGTTAAGGGCATTATCATTGTGAAGTTGGTAACAATGGACAAAAATGAGTATGTGCAAACATAATATTGATGTAAGTTGGTATCTATGAAAAGTTTGTAAATGTTATTATGAAAGCGACAGAAGAATTTCAAGTTGGTAATGGTCTTAATGAAGAATCTTTGCGTTAAACATATATTTTCAGGTACGTGTACAGACAACTTTGTGTGAATATGATCATGTTATTGATTTAATTTCTCATATACCCTTTAGACTCTGCCAAACTTTATCTCTATATATATTTAAATCCTGGACAATGATAGAATCTATTACAATG
