# Supplementary figures and images for: Ethylene promotes anthocyanin synthesis in ‘Viviana’ lily via the LvMYB5-LvERF113-LvMYB1 module
Source: Hortic Res. 2025 Feb 25;12(6):uhaf059. doi: 10.1093/hr/uhaf059 (PMC12023856; doi:10.1093/hr/uhaf059)

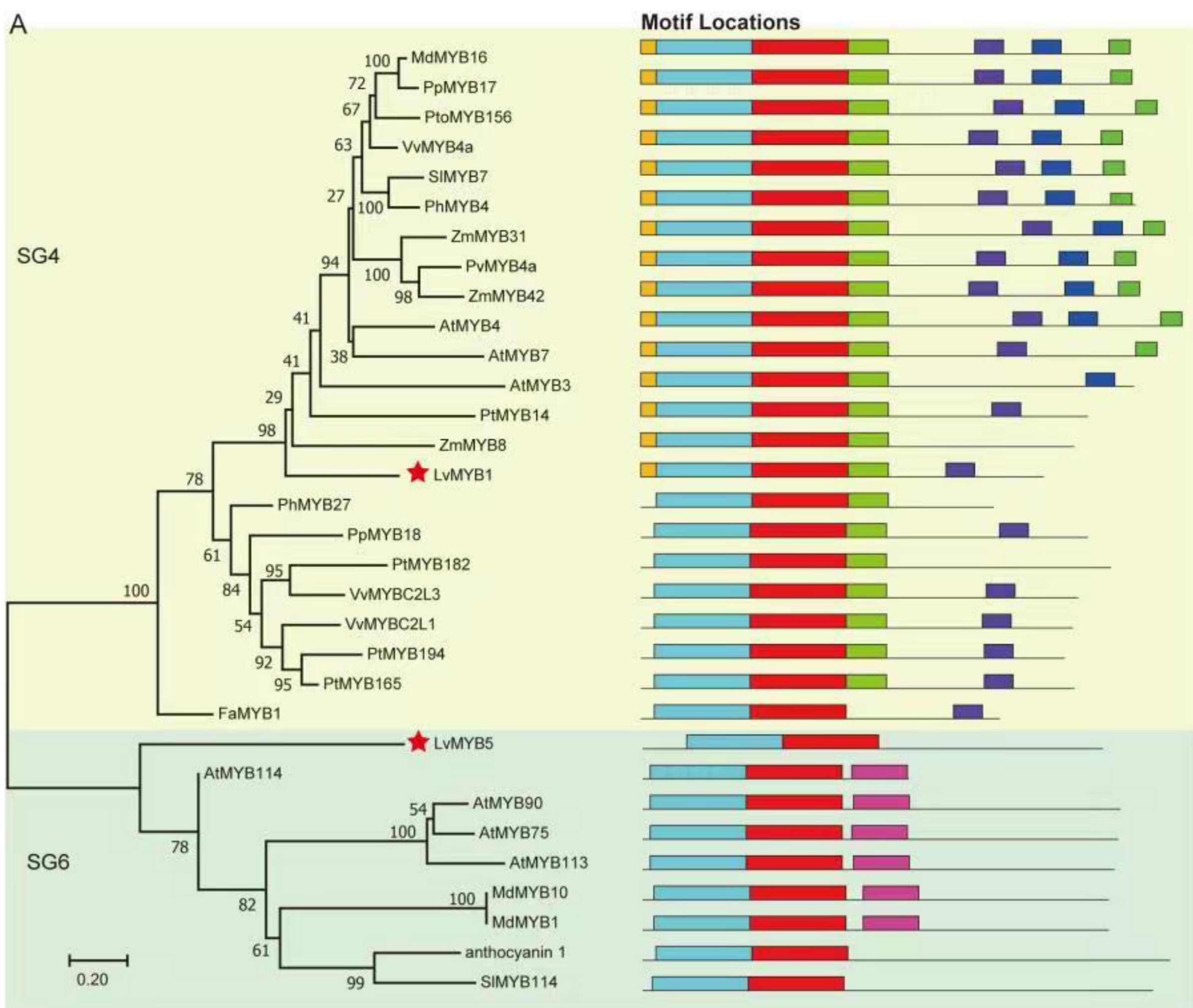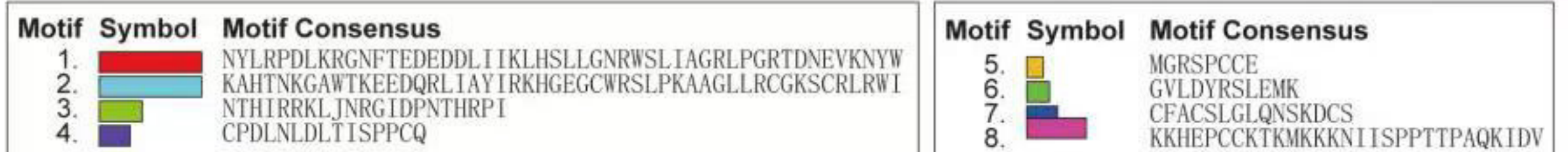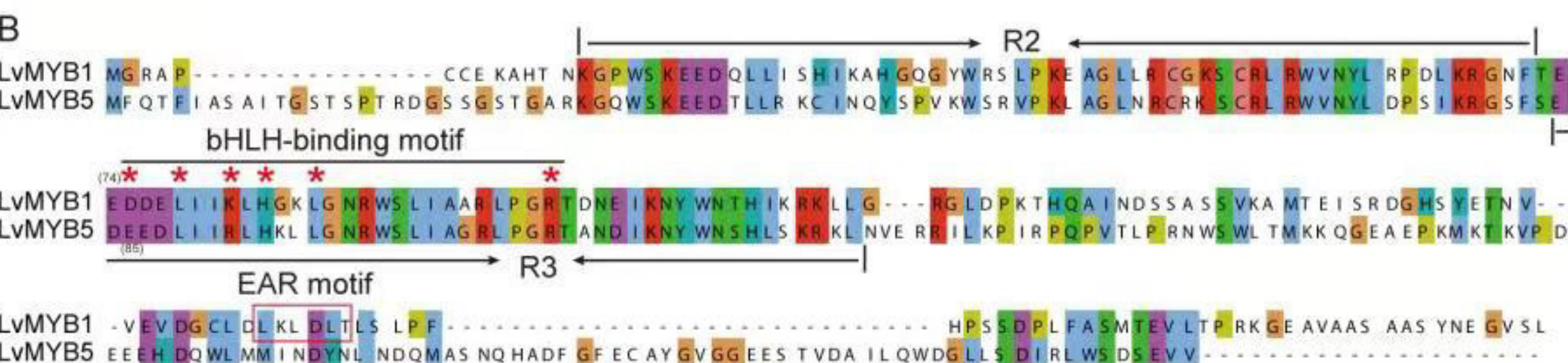

Supplement: Web_Material_uhaf059 [file web_material_uhaf059.zip › FigS1.pdf]
